# Low-Value Clinical Practices in Pediatric Trauma Care

**DOI:** 10.1001/jamanetworkopen.2024.40983

**Published:** 2024-10-29

**Authors:** Theony Deshommes, Gabrielle Freire, Natalie Yanchar, Roger Zemek, Marianne Beaudin, Antonia Stang, Matthew John Weiss, Sasha Carsen, Isabelle J. Gagnon, Belinda J. Gabbe, Melanie Bérubé, Henry Thomas Stelfox, Suzanne Beno, Melanie Labrosse, Emilie Beaulieu, Simon Berthelot, Terry Klassen, Alexis F. Turgeon, François Lauzier, Xavier Neveu, Amina Belcaid, Anis Ben Abdeljelil, Pier-Alexandre Tardif, Marianne Giroux, Lynne Moore

**Affiliations:** 1 Department of Social and Preventive Medicine, School of Medicine, Laval University, Québec City, Québec, Canada; 2 Population Health and Optimal Health Practices Research Unit, Trauma–Emergency–Critical Care Medicine, Centre de Recherche du CHU de Québec – Université Laval (Hôpital de l’Enfant-Jésus), Québec City, Québec, Canada; 3 Department of Pediatrics, Alpert Medical School of Brown University, Providence, Rhode Island; 4 Division of Emergency Medicine, Department of Pediatrics, Faculty of Medicine, University of Toronto, Toronto, Ontario, Canada; 5 Child Health Evaluative Sciences Program, Peter Gilgan Institute for Research and Learning, The Hospital for Sick Children, Toronto, Ontario, Canada; 6 Department of Surgery, Medicine and Community Health Sciences, O’Brien Institute for Public Health, University of Calgary, Calgary, Alberta, Canada; 7 Department of Pediatrics, Children’s Hospital of Eastern Ontario, Ottawa, Ontario, Canada; 8 Department of Pediatric Surgery, CHU Sainte-Justine, University of Montreal, Québec, Canada; 9 Department of Pediatrics, Emergency Medicine, and Community Health Sciences, Cumming School of Medicine, University of Calgary, Alberta, Canada; 10 Department of Pediatrics, School of Medicine, Laval University, Québec City, Québec, Canada; 11 Division of Pediatric Critical Care Medicine, Mère-Enfant Soleil hospital, Québec City, Québec, Canada; 12 Division of Orthopaedic Surgery, Children’s Hospital of Eastern Ontario, Ottawa, Ontario, Canada; 13 Division of Pediatric Emergency Medicine, McGill University Health Centre, Montreal Children’s Hospital, Montreal, Québec, Canada; 14 School of Physical and Occupational Therapy, Faculty of Medicine and Health Sciences, McGill University, Montreal, Québec, Canada; 15 School of Public Health and Preventive Medicine, Monash University, Melbourne, Victoria, Australia; 16 Faculty of Nursing, Université Laval, Québec City, Québec, Canada; 17 Departments of Critical Care Medicine, Medicine and Community Health Sciences, O’Brien Institute for Public Health, University of Calgary, Alberta, Canada; 18 Division of Emergency Medicine, The Hospital for Sick Children, University of Toronto, Toronto, Ontario, Canada; 19 Department of Pediatrics, Division of Emergency Medicine, CHU Sainte-Justine, Université de Montréal, Montreal, Québec, Canada; 20 Department of Pediatrics, Université Laval, Québec City, Québec, Canada; 21 George & Fay Yee Centre for Health Care Innovation, Children’s Hospital Research Institute of Manitoba, Department of Pediatrics and Child Health, University of Manitoba, Winnipeg, Manitoba, Canada; 22 Department of Anesthesiology and Critical Care Medicine, Université Laval, Québec City, Québec, Canada; 23 Institut National d’Excellence en Santé et Services Sociaux, Québec City, Québec, Canada

## Abstract

**Importance:**

Reducing low-value care has the potential to improve patient experiences and outcomes and decrease the unnecessary use of health care resources. Research suggests that low-value practices (ie, the potential for harm exceeds the potential for benefit) in adult trauma care are frequent and subject to interhospital variation; evidence on low-value practices in pediatric trauma care is lacking.

**Objective:**

To estimate the incidence of low-value practices in pediatric trauma care and evaluate interhospital practice variation.

**Design, Setting, and Participants:**

A retrospective multicenter cohort study in a Canadian provincial trauma system was conducted. Children younger than 16 years admitted to any of the 59 provincial trauma centers from April 1, 2016, to March 31, 2022, were included.

**Main Outcomes and Measures:**

Low-value practices were identified from systematic reviews of clinical practice guidelines on pediatric trauma. The frequencies of low-value practices were evaluated by estimating incidence proportions and cases per 1000 admissions (low if ≤10% and ≤10 cases, moderate if >10% or >10 cases, and high if >10% and >10 cases) were identified. Interhospital variation with intraclass correlation coefficients (ICCs) were assessed (low if <5%, moderate if 5%-20%, and high if >20%).

**Results:**

A total of 10 711 children were included (mean [SD] age, 7.4 [4.9] years; 6645 [62%] boys). Nineteen low-value practices on imaging, fluid resuscitation, hospital/intensive care unit admission, specialist consultation, deep vein thrombosis prophylaxis, and surgical management of solid organ injuries were identified. Of these, 14 (74%) could be evaluated using trauma registry data. Five practices had moderate to high frequencies and interhospital variation: head computed tomography in low-risk children (7.1%; 33 per 1000 admissions; ICC, 8.6%), pretransfer computed tomography in children with a clear indication for transfer (67.6%; 4 per 1000 admissions; ICC, 5.7%), neurosurgical consultation in children without clinically important intracranial lesions (11.6%; 13 per 1000 admissions; ICC, 15.8%), hospital admission in isolated mild traumatic brain injury (38.8%; 98 per 1000 admissions; ICC, 12.4%), and hospital admission in isolated minor blunt abdominal trauma (10%; 5 per 1000 admissions; ICC, 31%).

**Conclusions and Relevance:**

In this cohort study, low-value practices appeared to be frequent and subject to interhospital variation. These practices may represent priority targets for deimplementation interventions, particularly as they can be measured using routinely collected data.

## Key Points

**Question:**

Are low-value clinical practices (ie, the potential for harm exceeds the potential for benefit) in pediatric trauma care frequent and subject to interhospital variation?

**Findings:**

In this cohort study including 59 trauma centers, 14 low-value clinical practices in acute pediatric trauma care could be evaluated using trauma registry data. Of these, 5 practices pertaining to initial head imaging, pretransfer imaging, neurosurgical consultation, and hospital admission were frequent and varied across hospitals.

**Meaning:**

These practices may represent priority targets for deimplementation interventions, particularly as they can be measured using routinely collected data.

## Teaser

This cohort study examines low-value clinical practices, ie, those with the potential for harm exceeding the potential for benefit, in pediatric trauma centers.

## Introduction

Studies conducted in the US have estimated that low-value care, defined as the provision of medical services for which the potential for harm exceeds the potential for benefit,^1^ represents up to 33% of all health care spending.^2^ Low-value care exposes patients to negative physical, psychological, and financial consequences.^3,4,5^ The deimplementation of low-value clinical practices is integral to the delivery of high-quality care and the sustainability of health care systems.^6,7,8,9^

Injury is the leading cause of death and disability in North American children and represents more than 3 million hospitalizations per year in the US.^10^ Pediatric trauma is particularly vulnerable to low-value care because of the heterogeneity in clinical presentations across the spectrum of age and development, lack of experience and knowledge in treating pediatric trauma at nonpediatric centers, lack of robust evidence to support pediatric trauma practice, and centralization of expertise at very few urban trauma centers, increasing the need for transfers and communication issues.^11,12,13,14,15,16^ Research has identified low-value clinical practices in adult trauma care that are frequent, costly, and vary substantially across hospitals.^17,18,19,20^ However, evidence for pediatric trauma populations is lacking.^21^ This information would be valuable for benchmarking performance and identifying targets for quality improvement.^22^ We aimed to estimate the incidence of low-value practices in acute pediatric trauma care and evaluate interhospital practice variation.

## Methods

The protocol for this study was approved by all authors prior to analyses. The study was supported by a multidisciplinary advisory committee comprising pediatric emergency physicians, orthopedic/trauma and neurosurgeons, neurointensivists, nurse practitioners, rehabilitation specialists, emergency physicians in adult referral centers, trauma data specialists, and 3 parent partners. This project received approval from the CHU de Québec-Université Laval Research Ethics Board. Individual patient consent was not deemed necessary because data were deidentified for both patients and trauma centers. Results are reported according to the Strengthening the Reporting of Observational Studies in Epidemiology (STROBE) reporting guideline.^23^

### Study Design and Setting

We conducted a multicenter, retrospective cohort study based on the integrated trauma system in Québec, Canada. Acute care is provided in the 59 designated trauma centers, including 5 level I, of which 2 are pediatric; 5 level II; 21 level III; and 28 level IV centers.

### Population

We included all children younger than 16 years (age cutoff for transportation to a pediatric trauma center in the Québec system) with a primary diagnosis of injury who were admitted to any of the 59 provincial trauma centers between April 1, 2016, and March 31, 2022. Children with a primary diagnosis of burns, foreign objects, poisoning, drowning, or late effects of injury were excluded, as were children in cardiorespiratory arrest on arrival who were declared dead within 30 minutes.

### Data Sources

Data were extracted from the provincial trauma registry, with mandatory entry for all hospital admissions with a primary diagnosis of injury in all centers, regardless of severity. It is estimated that the database contains information on more than 90% of all major trauma that occurs in the province.^24^ Injury diagnoses are coded with the Abbreviated Injury Scale (AIS-2008 update) and interventions are coded with the *International Statistical Classification of Diseases and Related Health Problems, Tenth Revision* (*ICD-10*). Data quality mechanisms include standardized periodic training, discussion forums, monthly meetings dedicated to discussing coding queries, built-in coding algorithms, periodic data audits, and publication of data quality statistics.

### Identification of Low-Value Clinical Practices

We identified low-value clinical practices from five 2023 systematic reviews on clinical practice guidelines for pediatric trauma.^11,13,16,25,26^ To measure the incidence of the low-value practices, we developed coding algorithms by consulting the literature and advisory committee members (eTable 1 in Supplement 1). These algorithms included patient characteristics (age, comorbidities), anatomic injuries (AIS codes), physiologic parameters (Glasgow Coma Scale, systolic blood pressure, and heart rate), interventions (*ICD-10* codes), specialist consultations, intensive care unit (ICU) admission, and transfer from another acute care center. Coding algorithms were developed iteratively through cycles of derivation and case verification with clinical experts until no further changes were required.

### Statistical Analysis

First, we estimated incidence proportions with 95% CIs and calculated the number of cases per 1000 admissions for each practice. Based on the literature, frequencies were considered low if the incidence was less than or equal to 10% and there were less than or equal to 10 cases per 1000 admissions, moderate if the incidence was greater than 10% or there were more than 10 cases per 1000 admissions, and high if the incidence was greater than 10% and there were more than 10 cases per 1000 admissions.^18,27,28,29^ Second, to evaluate practice variation, we used logistic multilevel regression models with trauma centers entered as a random intercept and designation level (pediatric trauma center [PTC], adult level I/II, and adult level III/IV) entered as a fixed effect to calculate incidences by designation level and intraclass correlation coefficients (ICCs). The ICCs measure the proportion of the total variation in the practice explained by the hospital effect, interpreted as weak (ICC, <5%), moderate (ICC, 5%-20%), and strong (ICC, >20%).^18,30^

We conducted subgroup analyses according to age (<1, 1-5, 6-11, and 12-15 years), biological sex, and study period (April 2016 to March 2020; April 2020 to March 2022 to cover pre and peri/post pandemic periods). No information on gender or race and ethnicity was available in the dataset. All analyses were performed using SAS, version 9.4 (SAS Institute Inc).

## Results

### Low-Value Clinical Practices

We identified 19 recommendations on low-value clinical practices in pediatric trauma guidelines (Table). Among these, 11 practices (58%) pertained to diagnostic imaging, 1 to fluid resuscitation, 1 to specialist consultation, 2 to hospital admission, 1 to ICU admission, 2 to management of solid organ injuries, and 1 to deep vein thrombosis (DVT) prophylaxis. We developed and validated algorithms for 14 of 19 practices (74%). The 5 remaining practices could not be measured with data from the trauma registry.

**Table.** Low-Value Practices for Acute Pediatric Trauma Care Identified in Clinical Practice Guidelines

Low-value practice	Clinical practice guideline
**Evaluated using trauma registry data**	
Head CT in children at low risk on a validated clinical decision rule	PECARN, NEXUS, CHALICE, CDC, CBO, CW, SNC, ACR, NICE
Cervical spine CT in children at low risk on a validated clinical decision rule	PECARN, NEXUS, CDC, CBO, CW, SNC, ACR, NICE
Abdominal/pelvic CT in children at low risk on a validated clinical decision rule	PECARN, CDC, CBO, CW, SNC, ACR, NICE
Use of whole-body CT in children	NICE
Pretransfer CT in children with a clear indication for transfer	ISPEM, TREKK
Posttransfer repeat CT in children without clinical deterioration	BTF, TREKK, ISPEM, ACS, CWC, BIG Kids
Repeat head CT in children without clinically important intracranial lesions or clinical deterioration	CW, BIG Kids, NICE
Neurosurgical consultation in children without clinically important intracranial lesions	BIG Kids
Hospital admission in children with isolated mild TBI at low risk on a validated clinical decision rule	BIG Kids
Hospital admission in children with isolated blunt abdominal trauma, normal physical examination, asymptomatic, and negative FAST or CT results	BIG Kids
ICU admission in children with isolated head injury and no clinically important intracranial lesions	BIG Kids
Surgical management in children with solid organ injury who are hemodynamically stable	EAST/PTS, WSES
DVT prophylaxis in prepubertal children	EAST
Angiointervention in hemodynamically stable children with low grade (I-III) solid organ injury	APSA
**Could not be evaluated using trauma registry data**	
Use of FAST in addition to abdominal CT	NICE
Use of crystalloids in the ED	NICE, ERC
Skull radiographs for children with TBI	CDC, ACR
Wrist radiographs in children with a wrist injury, aged >2 y, and normal physical examination	ACR
Ankle radiographs in children aged >2 y that are negative on a validated clinical decision rule	ACR

Abbreviations: ACR, American College of Radiology; ACS, American College of Surgeons; APSA, American Pediatric Surgery Association; BIG, Brain Injury Guidelines; BTF, Brain Trauma Foundation; CBO, Dutch Institute for Health Care Improvement; CDC, Centers for Disease Control and Prevention; CHALICE, Children’s Head Injury Algorithm for the Prediction of Important Clinical Events; CT, computed tomography; CW, Choosing Wisely; DVT, deep vein thrombosis; EAST, Eastern Association for the Surgery of Trauma/Pediatric Trauma Society; ED, emergency department; ERC, European Resuscitation Council; FAST, focused assessment with sonography in trauma; ICU, intensive care unit; ISPEM, Italian Society of Pediatric Emergency Medicine; NEXUS, National Emergency X-Radiography Utilization Study; NICE, National Institute for Health and Care Excellence; PECARN, Pediatric Emergency Care Applied Research Network; PTS, Pediatric Trauma Society; SNC, Scandinavian Neurotrauma Committee; TBI, traumatic brain injury; TREKK, Translating Emergency Knowledge for Kids; WSES, World Society of Emergency Surgery.

### Study Population

Between April 1, 2016, and March 31, 2022, 10 711 patients younger than 16 years were admitted to a trauma center in Québec with a primary diagnosis of injury. Our study sample comprised 1072 (10%) children younger than 1 year, 2248 (21%) aged 1 to 4 years, 4434 (41%) aged 5 to 11 years, and 2957 (28%) aged 12 to 15 years (mean [SD] age, 7.4 [4.9] years; 4066 [38%] girls; 6645 [62%] boys) (eTable 2 in Supplement 1). The most frequent mechanisms of injury were falls (6469 [60%]), blunt object trauma (2830 [26%]), and motor vehicle crashes (1191 [11%]). Nonaccidental injuries accounted for 3% of all admissions, but 13% of these were in children younger than 1 year. Forty-two percent of children received definitive treatment in a PTC and 39% in a level III center. The most common injury location was the extremities (60%), except in children younger than 1 year who most frequently had head injuries (79%). Most children were admitted with minor injuries; 94% had an Injury Severity Score less than or equal to 12 and 79% had a maximum AIS score less than 3. Among children with documented physiologic parameters on arrival in the emergency department (ED), 85% were hemodynamically stable and 96% had a Glasgow Coma Scale score greater than or equal to 13. Fifty percent of the children were admitted to an orthopedic department, 23% to a general pediatrics department, and 13% to a trauma ward. Most patients (97%) were discharged home with (58%) or without (39%) ambulatory follow-up.

### Incidence of Low-Value Practices

**Figure 1.**
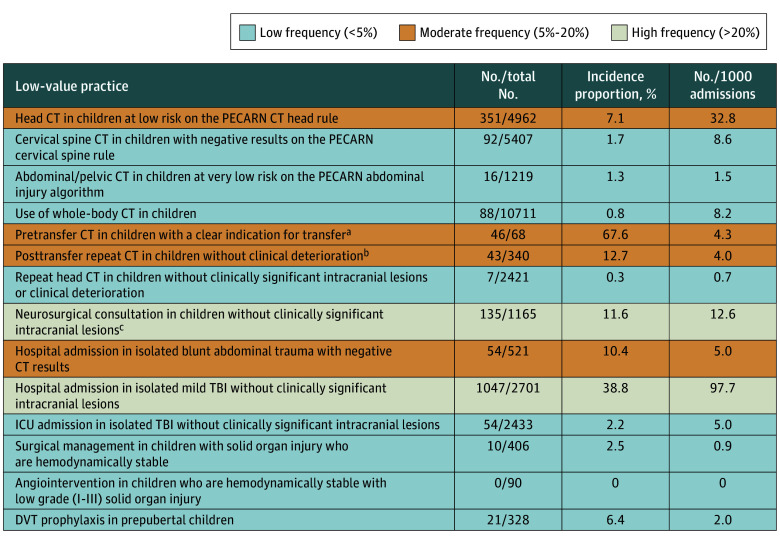
Relative (Incidence Proportions) and Absolute (Case Volumes) Frequencies of Low-Value Practices in Pediatric Trauma Admissions, 2016-2022 CT indicates computed tomography; DVT, deep vein thrombosis; ICU, intensive care unit; PECARN, Pediatric Emergency Care Applied Research Network; and TBI, traumatic brain injury. ^a^Applies to level III/IV referral centers. ^b^Applies to pediatric trauma centers and level I/II adult trauma centers. ^c^Consultation in the emergency department for neurosurgical centers (level I /II) and transfer to neurotrauma trauma centers for level III/IV centers.

**Figure 2.**
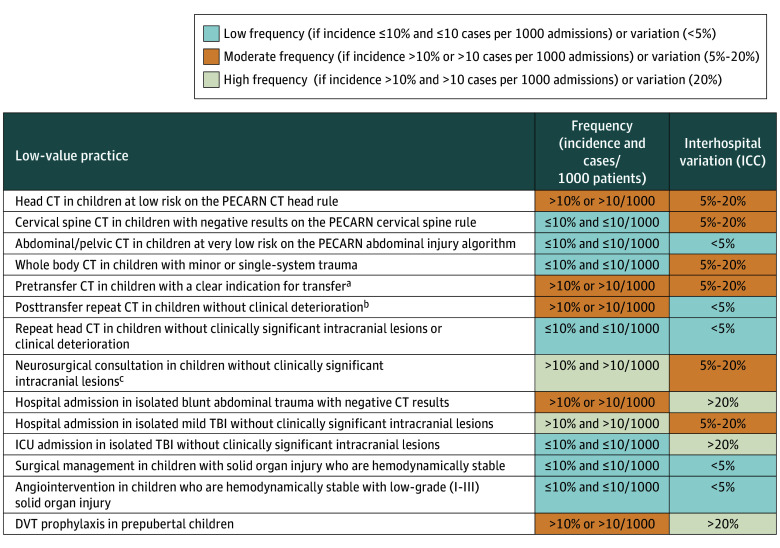
Summary of Relative and Absolute Frequencies and Interhospital Variation of Low-Value Practices in Pediatric Trauma Admissions, 2016-2022 CT indicates computed tomography; DVT, deep vein thrombosis; ICC, intraclass correlation coefficient; ICU intensive care unit; PECARN, Pediatric Emergency Care Applied Research Network; and TBI, traumatic brain injury. ^a^Applies to level III/IV referral centers. ^b^Applies to pediatric trauma centers and level I/II adult trauma centers. ^c^Consultation in the emergency department for neurosurgical centers (level I/ II) and transfer to neurotrauma trauma centers for level III/IV centers.

All low-value practices related to initial diagnostic imaging had relative frequencies below 10% for head computed tomography (CT) (7.1%), cervical spine CT (1.7%), abdominal/pelvic CT (1.3%), and whole-body CT (0.8%) (Figure 1 and Figure 2). However, head CT in low-risk patients had a high absolute frequency (33 cases per 1000 admissions). Pretransfer imaging and posttransfer repeat imaging had high relative frequencies (pretransfer, 68%; posttransfer, 13%), but low absolute frequencies (pretransfer, 4.3 cases per 1000 admissions; posttransfer, 4 cases per 1000 admissions). Neurosurgical consultation in children without clinically important intracranial lesions had high relative (12%) and absolute (13 per 1000 admissions) frequencies, as did hospital admission for isolated mild traumatic brain injury (TBI) (39%, 98 per 1000 admissions). Hospital admission for isolated minor blunt abdominal trauma had high relative but low absolute frequency (10%, 5 per 1000 admissions). Low relative and absolute frequencies were seen for ICU admission in isolated TBI without clinically important intracranial lesions (2.2%, 5 per 1000 admissions), surgical management (2.5%, 0.9 per 1000 admissions), and angiointervention (0%, 0 per 1000 admissions) in hemodynamically stable children with solid organ injuries, and DVT prophylaxis in prepubertal children (6.4%, 2.0 per 1000 admissions).

### Interhospital Variation

**Figure 3.**
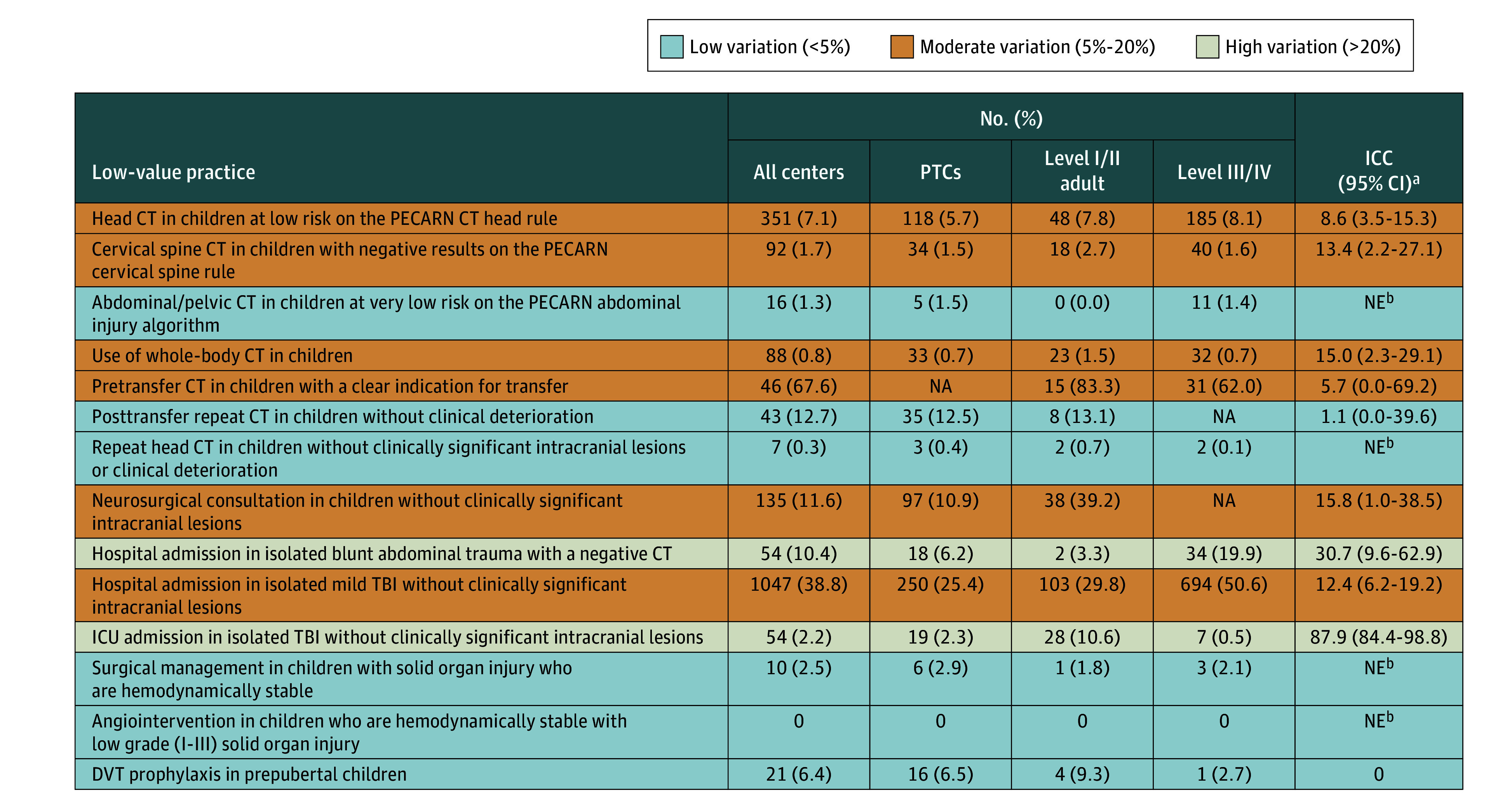
Interhospital Variation in Low-Value Practices for Pediatric Trauma Admissions, 2016-2022 CT indicates computed tomography; DVT, deep vein thrombosis; ICC, intraclass correlation coefficient; ICU, intensive care unit; NA, not applicable; NE, not estimated; PECARN, Pediatric Emergency Care Applied Research Network; PTC, pediatric trauma center; and TBI, traumatic brain injury. ^a^Interpreted as low (<5%), moderate (5%-20%), and high (>20%). ^b^Could not be estimated due to low sample sizes but considered to be low as numbers were low in all centers.

In comparisons across trauma center designation levels, PTCs had lower incidences of low-value head (5.7%), cervical spine (1.5%), and whole-body (0.7%) CT than adult trauma level I/II centers (head, 7.8%; cervical spine, 2.7%; whole-body, 1.5% CT) or level III/IV centers (head, 8.1%; cervical spine, 1.6%; whole-body, 1.4% CT) (Figure 3). Similarly, PTCs had lower incidences of hospital admission for mild TBI (6%) or minor abdominal trauma (25%) than level III/IV centers (mild TBI, 20%; minor abdominal trauma, 51%). Furthermore, in PTCs, lower incidences of neurosurgical consultation (11%) and ICU admission (2%) for children without clinically important intracranial lesions, as well as DVT prophylaxis (6.5%), were observed than in adult trauma centers (neurosurgical consultation, 39%; ICU admission, 11%; DVT prophylaxis, 9.3%). Interhospital variation was high (ICC, >20%) for hospital admission in isolated minor blunt abdominal trauma (31%) and ICU admission for isolated TBI without clinically important intracranial lesions (88%) (Figure 2 and Figure 3). Moderate interhospital variation was observed for head CT (9%), cervical spine CT (13%), whole-body CT (15%), pretransfer CT (6%), neurosurgical consultation (16%), and hospital admission in isolated mild TBI (12%). Interhospital variation was low for posttransfer CT and DVT prophylaxis and could not be estimated due to low patient volumes for abdominal/pelvic CT, repeat head CT, and surgery/angiointervention for solid organ injuries.

### Subgroup Analyses

Results varied little by biological sex and admission year, but we observed some heterogeneity over age groups (eTable 3 in Supplement 1). Specifically, children younger than 1 year had higher incidences of initial head CT in low-risk patients (23% and 89 per 1000 admissions vs 5% and 25 per 1000 admissions for other age groups). Similarly, neurosurgical consultations in the absence of clinically important intracranial lesions were more frequent in children younger than 1 year (18% and 43 per 1000 admissions vs approximately 10% and 10 per 1000 admissions for other age groups), and the incidence of hospital admission for minor blunt abdominal trauma decreased with age (<1 year, 44%; 1-4 years, 18%; 5-11 years, 11%; and 12-15 years, 5%).

## Discussion

We identified 19 recommendations pertaining to low-value clinical practices in acute pediatric trauma care. Of these, we were able to measure 14 (74%) with trauma registry data. Head CT in children at low risk on a clinical decision rule, pretransfer CT in children with a clear indication for transfer, neurosurgical consultation for TBI without clinically important intracranial lesions, hospital admission in isolated mild TBI, and hospital admission in isolated minor blunt abdominal trauma all had moderate to high frequencies and interhospital variation. Frequencies were low, but interhospital variation was moderate to high for whole-body CT and ICU admission for isolated TBI without clinically important intracranial lesions. Frequencies and interhospital variation were low for initial abdominal/pelvic CT, repeat head CT in children without clinically important intracranial lesions or clinical deterioration, surgical management or angiointervention for hemodynamically stable children with solid organ injury, and DVT prophylaxis in prepubertal children. In general, low-value practices were less frequent in PTCs than other trauma centers. Low-value head CT, neurosurgical consultation, and hospital admission in minor blunt abdominal trauma were more frequent for younger children, but varied little by biological sex or time period.

Three of the 5 low-value practices with high frequency and interhospital variation were related to the treatment of mild TBI (ie, imaging, neurosurgical consultation, and hospital admission). Persistent overtreatment and overtriage of children with mild TBI, despite the widespread implementation of validated clinical decision rules,^31,32^ has been reported elsewhere.^33,34^ It may be explained by fear of missing clinically important intracranial lesions including the potential medicolegal repercussions, desire to relieve parent anxiety, or discomfort diagnosing, treating, or observing children in nonpediatric hospitals due to ED overcrowding or lack of pediatric readiness.^15^ The latter could also explain why these low-value practices were more common in level III/IV centers than in PTCs. Intensive care unit admission for isolated TBI without clinically important intracranial lesions was uncommon but had very high interhospital variation, with a high incidence in level I/II adult centers. This could be explained by a lack of ICU beds in PTCs or discomfort in monitoring children with mild complicated TBI outside the ICU setting in adult centers. The frequency of repeat head CT in children without clinically important intracranial lesions or clinical deterioration was low, unlike in a previous report.^35^ This may be due to data availability issues, whereby only the first CT in each body region in referral centers, the ED, and after admission are recorded in our trauma registry, likely leading to underestimation. Implementation of the Brain Injury Guidelines algorithm, now validated for pediatric populations, may facilitate the deimplementation of low-value practices in patients with mild TBI.^31,36^

Like findings in mild TBI, hospital admissions in isolated minor abdominal trauma had moderate frequency and high interhospital variation, with a much higher frequency in level III/IV trauma centers than in PTCs. Given that 78% of our pediatric trauma population admitted to the hospital had a maximum AIS of less than or equal to 2, we suspect that these findings represent overtriage, which has been reported previously for pediatric trauma populations and other health care conditions in children.^37,38^ Overtriage is likely caused by a lack of confidence in examination skills and/or lack of resources to provide an adequate period of observation in the ED, again related to pediatric readiness.^12,15^ Twenty-five percent of these patients were transferred in, leading to considerable additional resource use. Overtriage is a major problem in trauma systems, especially for pediatric patients, who often travel over large geographic distances to a PTC, putting a major strain on limited health care resources and generating a substantial financial and psychological burden for children and their families.^39,40^

The high incidence of imaging in level III/IV centers prior to transfer is also likely to be due to a lack of pediatric readiness, generating doubts about the appropriateness of transfer, a desire to provide a more precise/accurate report of injuries before speaking to the referral center, or fear of medicolegal repercussions if they miss an injury and the patient’s condition deteriorates in transport.^12,15^ Repeat posttransfer CT in PTCs was also above 10%, likely reflecting technological challenges in transferring images or radiology reports, as well as a lack of adequate imaging protocols in referral centers leading to insufficient image quality.^41^ Frequencies of initial diagnostic imaging in body regions other than the head were low, suggesting better adherence to clinical practice guideline recommendations and uptake of the as low as reasonably achievable concept on pediatric imaging.^42^ However, interhospital variation was moderate for whole-body CT, suggesting that this practice may be a target for knowledge translation efforts to further reduce its use, given the consequences in terms of radiation-associated cancer.^42^ The low incidence of surgical management and angiointervention for pediatric solid organ injuries has been reported elsewhere^20,43^ and suggests that adherence to guidelines published by surgical organizations is high, despite the lack of strong, evidence-based recommendations on surgical trauma care specific to pediatric populations.^16^ Similarly, low use of DVT prophylaxis in younger children suggests good adherence to guidelines^44^ that are based on a risk of venous thromboembolism of only 0.1% in children aged 12 years or younger following trauma.^45^

In subgroup analyses, the higher frequency of low-value initial head CT in children younger than 1 year likely reflects the application of guidelines for suspected nonaccidental injury in which radiography skeletal survey with head CT is recommended.^46^ Neurosurgical consultation and hospital admission for minor abdominal trauma were also more common in younger age groups, likely reflecting greater discomfort with examination, diagnosis, and monitoring of preverbal children in nonpediatric centers.^12,15^ The lack of difference in results over time suggests that the COVID-19 pandemic had minimal influence on the incidence of low-value pediatric trauma care.

### Strengths and Limitations

This study fills an important knowledge gap on low-value practices in pediatric trauma care. We had a large sample including all hospital admissions to all provincial trauma centers during 7 years, representing approximately 90% of major trauma treated in our province.^24^ However, several limitations need to be considered when interpreting the results. First, there is a risk that low-value clinical practices were misclassified because of data quality issues or lack of granular data in the trauma registry. For example, data on hemodynamic stability and the Glasgow Coma Scale were only recorded on ED arrival and were missing for 12% of children at referring and definitive care centers and for 40% of children on hospital admission in the definitive care centers. Previous work on missing data in our trauma registry suggests that these data are more likely to be missing in patients with minor extracranial injuries, but this may have led to an overestimation of the use of some low-value practices. Furthermore, misclassification bias may be differential if coding practices differ across centers, which may have led to an overestimation of interhospital variation. Lack of clinical information in the trauma registry meant that we could not develop measures for 5 of 19 identified low-value practices. Second, given that our measures of low-value practices were based on carefully selected denominators of patients who should not have received the practice, we did not adjust for patient case mix. However, since measurement error cannot be ruled out, residual confounding in interhospital comparisons may be present in our findings. Third, assessments of low-value initial diagnostic imaging would be more comprehensive if they were based on all ED presentations rather than hospital admissions. However, most ED data sources do not contain sufficient clinical information to identify low-value imaging accurately. Furthermore, we anticipate that hospitals with high rates of imaging in the ED would be identified using data on hospitalized patients. Fourth, despite having a province-wide sample, volumes were too low to assess interhospital variation for abdominal/pelvic CT, repeat head CT, and surgery/angiointervention in solid organ injuries. However, low relative and absolute frequencies suggest they these practices are unlikely to represent meaningful targets for deimplementation. Fifth, different contextual factors that are not documented in the trauma registry, such as clinical symptoms, social concerns, or local resource policies, likely explain the use of some of these practices.

Overall, limitations in data availability and the quality of evidence, in addition to the role of physician experience and parent/patient preference, mean that use of these practices on a patient level may well have been appropriate and a 0% incidence should not be the goal. Rather, interhospital variations should be seen as opportunities for quality improvement and any deimplementation intervention should be preceded by case revision to evaluate whether care was indeed low value.

## Conclusions

In this multicenter, retrospective cohort study, we identified 5 clinical practices in acute pediatric trauma care that are frequent and vary across trauma centers pertaining to initial head CT, pretransfer CT, neurosurgical consultation, and hospital admission. These practices may represent priority targets for deimplementation interventions, particularly as they can be measured using routinely collected data. Multifaceted interventions have been shown to be most useful to improve health care quality and could be based on existing clinical decision rules, shared decision-making tools, trauma readiness checklists, and quality metrics. However, such interventions should consider barriers to deimplementation, which are likely to differ across settings.

## Notes

Supplement 1. **eTable 1.** Metrics Used to Identify Low-Value Practices **eTable 2.** Characteristics of Pediatric Trauma Admissions, 2016-2022 **eTable 3.** Subgroup Analyses: Relative and Absolute Frequencies and Inter-Hospital Variation of Low-Value Practices by Age Group, Biological Sex, and Admission Year for Pediatric Trauma Admissions, 2016-2022

Supplement 2.
Data Sharing Statement
