# Supplementary material for: Low-Value Clinical Practices in Pediatric Trauma Care
Source: JAMA Netw Open. 2024 Oct 29;7(10):e2440983. doi: 10.1001/jamanetworkopen.2024.40983 (PMC11522939; doi:10.1001/jamanetworkopen.2024.40983)
Supplement: Supplement 1. — eTable 1. Metrics Used to Identify Low-Value Practices eTable 2. Characteristics of Pediatric Trauma Admissions, 2016-2022 eTable 3. Subgroup Analyses: Relative and Absolute Frequencies and Inter-Hospital Variation of Low-Value Practices by Age Group, Biological Sex, and Admission Year for Pediatric Trauma Admissions, 2016-2022 [file jamanetwopen-e2440983-s001.pdf]

Supplementary Online Content

Deshommes T, Freire G, Yanchar N, et al. Low-value clinical practices in pediatric trauma care. *JAMA Netw Open*. 2024;7(10):e2440983. doi:10.1001/jamanetworkopen.2024.40983**.**

eTable 1. Metrics Used to Identify Low-Value Practices

eTable 2. Characteristics of Pediatric Trauma Admissions, 2016-2022

eTable 3. Subgroup Analyses: Relative and Absolute Frequencies and Inter-Hospital Variation of Low-Value Practices by Age Group, Biological Sex, and Admission Year for Pediatric Trauma Admissions, 2016-2022

This supplementary material has been provided by the authors to give readers additional information about their work.

© 2024 Deshommes T et al. *JAMA Network Open.*

eTable 1. Metrics used to identify low-value practices

Low-value practice	Denominator	Numerator
Head CT in children at low risk on the PECARN CT head rule	Hemodynamically stable children (below SIPA cutoffs and no blood product transfusions in the ED) with GCS=15, minor trauma (ISS<12), no intracranial lesions, no severe facial trauma (AIS>2), and none of the following: altered mental status, penetrating trauma, basal skull fracture, high-impact mechanism, retrograde amnesia, loss of consciousness, cephalie, vomiting	With a head CT
Cervical spine CT in children negative on the PECARN cervical spine rule	Hemodynamically stable children (below SIPA cutoffs and no blood product transfusions in the ED) with GCS=15, minor trauma (ISS<12), no spinal lesions or neck injury and none of the following: altered mental status, penetrating trauma, high-impact mechanism, acute paralysis, pre-existing vertebral pathologies	With a cervical spine CT
Abdominal/pelvic CT in children at very low risk on the PECARN abdominal injury algorithm	Hemodynamically stable children (below SIPA cutoffs and no blood product transfusions in the ED) with GCS>13, minor trauma (ISS<12), no significant abdominal/pelvic injuries (AIS>1) and none of the following: penetrating trauma, high-impact mechanism, vomiting	With an abdominal/pelvic CT in the ED
Use of whole-body CT in children	Children admitted to hospital following injury	With a whole-body CT
Pretransfer CT in children with a clear indication for transfer[a]	Hemodynamically children (below SIPA cutoffs and no blood product transfusions in the ED) with moderate to severe TBI (GCS<9) or spinal cord injury transferred to a pediatric trauma center or level I/II adult trauma center	With a CT in the referral hospital
Post-transfer repeat CT in children without clinical deterioration[b]	Children transferred to a major trauma center who had a CT in the referral center and have no clinical deterioration (GCS and SIPA remain stable)	With a repeat CT in the receiving hospital in the same body region on the same day
Repeat head CT in children without clinically significant intracranial lesions or clinical deterioration	Children with GCS 13-15 and none of the following: anticoagulation, EDH, IVH, displaced skull fracture, SDH≥8mm, IPH≥8mm, SAH bi-hemospheric or >3mm (pBIG=1) and no clinical deterioration (GCS and SIPA remain stable)	With a repeat head CT
Neurosurgical consultation in children without clinically significant intracranial lesions[c]	Children with GCS 13-15 and none of the following: anticoagulation, EDH, IVH, displaced skull fracture, SDH≥8mm, IPH≥8mm, SAH bi-hemospheric or >3mm (pBIG=1)	With a neurosurgical consultation
Hospital admission in isolated blunt abdominal trauma with negative CT	Children with isolated abdominal injury (no injuries with AIS≥1 in other body regions)	Who are hemodynamically stable (SIPA=) with minor abdominal injury (AIS=1) and no documented non-accidental trauma
Hospital admission in isolated mild TBI without clinically significant intracranial lesions	Children with isolated head injury (no injuries with AIS≥1 in other body regions)	With GCS 13-15 and none of the following: anticoagulation, EDH, no IVH, no displaced skull fracture, no SDH≥8mm, no IPH≥8mm, no SAH bi-hemospheric or >3mm† (pBIG=1), documented non-accidental trauma
ICU admission in isolated TBI without clinically	Children with GCS 13-15 and none of the following: anticoagulation, EDH, IVH, displaced skull fracture, SDH≥8mm, IPH≥8mm, SAH bi-hemospheric or >3mm,	Admitted to the ICU

© 2024 Deshommes T et al. *JAMA Network Open.*

significant intracranial lesions	major injuries (AIS≥3) in other body regions, requiring mechanical ventilation	
Surgical management in children with solid organ injury who are hemodynamically stable	Hemodynamically stable children (below SIPA cutoffs and no blood product transfusions in the ED) with any grade solid organ injury (spleen, kidney, liver) and no peritonitis	With a laparotomy within 6h of arrival
Angiointervention in hemodynamically stable children with low grade (I-III) solid organ injury	Hemodynamically stable children (below SIPA cutoffs and no blood product transfusions in the ED) with grade I-III solid organ injury (spleen, kidney, liver)	With an angiointervention
DVT prophylaxis in prepubertal children	Children aged ≤10 years with major trauma (ISS>12)	Received DVT prophylaxis

AIS, Abbreviated Injury Scale; pBIG: pediatric Brain Injury Guidelines; CT, computed tomography; ED, emergency department; EDH, epidural hematoma; GCS, Glasgow Coma Scale; ICU intensive care unit; IPH, intraparenchymal haemorrhage; ISS, Injury Severity Score; IVH, intraventricular hemorrhage; PECARN, Pediatric Emergency Care Applied Research Network; SAH, subarachnoid hemorrhage; SBP, systolic blood pressure; SDH, subdural hemorrhage; SIPA, Shock Index Pediatric Adjusted; TBI, traumatic brain injury

[a]Applies to level III and IV referral centers

[b]Applies to pediatric trauma centers and level I-II adult trauma centers

[c]Consultation in the emergency department for neurosurgical centers (level I and II) and transfer to neurotrauma trauma centers for level III/IV centers

© 2024 Deshommes T et al. *JAMA Network Open.*

eTable 2. Characteristics of pediatric trauma admissions, 2016-2022

N (%)	<1 y-o	1-4 y-o	5-11 y-o	12-15 y-o	Total
**All patients**	1072 (10.0)	2248 (21.0)	4434 (41.4)	2957 (27.6)	10711 (100.0)
**Year of admission**					
Apr 2016 – Mar 2018	407 (38.0)	801 (35.6)	1587 (35.8)	1028 (34.8)	407 (38.0)
Apr 2018 – Mar 2020	337 (31.4)	710 (31.6)	1469 (33.1)	979 (33.1)	337 (31.4)
Apr 2020 – Mar 2022	328 (30.6)	737 (32.8)	1378 (31.1)	950 (32.1)	328 (30.6)
**Female sex**	438 (45.1)	951 (42.9)	1842 (42.0)	835 (28.7)	4066 (38.8)
**Mechanism of injury**					
Motor vehicle collision	14 (1.3)	136 (6.0)	409 (9.2)	632 (21.4)	1191 (11.1)
Falling from own height	344 (32.1)	722 (32.1)	667 (15.0)	368 (12.4)	2101 (19.6)
Fall from height	405 (37.8)	850 (37.8)	2255 (50.9)	858 (29.0)	4368 (40.8)
Struck by a blunt object	303 (28.3)	486 (21.6)	1027 (23.2)	1014 (34.3)	2830 (26.4)
Penetrating	6 (0.6)	54 (2.4)	76 (1.7)	85 (2.9)	221 (2.1)
**Intent**[a]					
Unintentional	931 (86.8)	2197 (97.7)	4415 (99.6)	2884 (97.5)	10427 (97.3)
Non-accidental	143 (13.3)	53 (2.4)	21 (0.5)	84 (2.8)	301 (2.8)
Self-harm	0 (0.0)	1 (0.0)	5 (0.1)	27 (0.9)	33 (0.3)
**Trauma center designation level**					
Level I pediatric	481 (44.9)	1064 (47.3)	1858 (41.9)	1062 (35.9)	4465 (41.7)
Level I adult	2 (0.2)	0 (0.0)	5 (0.1)	102 (3.4)	109 (1.0)
Level II	131 (12.2)	238 (10.6)	628 (14.2)	439 (14.8)	1436 (13.4)
Level III	391 (36.5)	830 (36.9)	1765 (39.8)	1223 (41.4)	4209 (39.3)
Level IV	67 (6.3)	116 (5.2)	178 (4.0)	131 (4.4)	492 (4.6)
**Transfer-in**	231 (21.5)	608 (27.0)	1153 (26.0)	712 (24.1)	2704 (25.2)
**Any comorbidity**	15 (1.4)	75 (3.3)	163 (3.7)	210 (7.1)	463 (4.3)
**Body region of the worst injury**					
Head	841 (78.5)	985 (43.8)	981 (22.1)	577 (19.5)	3384 (31.6)
Thorax	30 (2.8)	44 (2.0)	72 (1.6)	98 (3.3)	244 (2.3)
Abdomen	9 (0.8)	67 (3.0)	239 (5.4)	206 (7.0)	521 (4.9)
Spine	2 (0.2)	27 (1.2)	62 (1.4)	95 (3.2)	186 (1.7)
Upper Extremities	75 (7.0)	668 (29.7)	2399 (54.1)	879 (29.7)	4021 (37.5)
Lower Extremities	115 (10.7)	457 (20.3)	681 (15.4)	1102 (37.3)	2355 (22.0)
**Maximum Abbreviated Injury Scale score**					
≤ 1	385 (35.9)	785 (34.9)	853 (19.2)	465 (15.7)	2488 (23.2)
2	386 (36.0)	910 (40.5)	2891 (65.2)	1759 (59.5)	5946 (55.5)
3	257 (24.0)	486 (21.6)	589 (13.3)	570 (19.3)	1902 (17.8)
4	26 (2.4)	38 (1.7)	66 (1.5)	104 (3.5)	234 (2.2)
≥ 5	18 (1.7)	29 (1.3)	35 (0.8)	59 (2.0)	141 (1.3)
**Injury Severity Score**					
≤ 8	761 (71.0)	1685 (75.0)	3717 (83.8)	2179 (73.7)	8342 (77.9)
9-12	218 (20.3)	469 (20.9)	537 (12.1)	485 (16.4)	1709 (16.0)
13-15	32 (3.0)	18 (0.8)	56 (1.3)	81 (2.7)	187 (1.7)
16-24	38 (3.5)	37 (1.6)	74 (1.7)	124 (4.2)	273 (2.5)
≥ 25	23 (2.1)	39 (1.7)	50 (1.1)	88 (3.0)	200 (1.9)
**Glasgow Coma Score**					
3-8	7 (0.7)	39 (1.7)	45 (1.0)	69 (2.3)	160 (1.5)

© 2024 Deshommes T et al. *JAMA Network Open.*

9-12	14 (1.3)	22 (1.0)	39 (0.9)	22 (0.7)	97 (0.9)
13-15	807 (75.3)	1446 (64.3)	2391 (53.9)	1560 (52.8)	6204 (57.9)
Missing	244 (22.8)	741 (33.0)	1959 (44.2)	1306 (44.2)	4250 (39.7)
**Hemodynamic stability[b]**					
Stable	957 (89.3)	1627 (72.4)	3328 (75.1)	2101 (71.1)	8013 (74.8)
Unstable	16 (1.5)	421 (18.7)	567 (12.8)	408 (13.8)	1412 (13.2)
Missing	99 (9.2)	200 (8.9)	539 (12.2)	448 (15.2)	1286 (12.0)
**Admission service**					
Orthopedics	64 (6.0)	934 (41.5)	2701 (60.9)	1687 (57.1)	5386 (50.3)
Pediatrics	673 (62.8)	757 (33.7)	686 (15.5)	389 (13.2)	2505 (23.4)
Trauma	223 (20.8)	229 (10.2)	524 (11.8)	385 (13.0)	1361 (12.7)
General surgery	3 (0.3)	25 (1.1)	127 (2.9)	154 (5.2)	309 (2.9)
Plastic surgery	5 (0.5)	73 (3.2)	91 (2.1)	55 (1.9)	224 (2.1)
Neurosurgery	66 (6.2)	35 (1.6)	54 (1.2)	55 (1.9)	210 (2.0)
Others	38 (3.5)	195 (8.7)	251 (5.7)	232 (7.8)	716 (6.7)
**Discharge Destination**					
Death	7 (0.7)	8 (0.4)	13 (0.3)	18 (0.6)	46 (0.4)
Home without follow-up	558 (52.1)	950 (42.3)	1576 (35.5)	1053 (35.6)	4137 (38.6)
Home with ambulatory follow up	460 (42.9)	1222 (54.4)	2747 (62.0)	1750 (59.2)	6179 (57.7)
Acute care	40 (3.7)	54 (2.4)	77 (1.7)	83 (2.8)	254 (2.4)
Rehabilitation	7 (0.7)	14 (0.6)	21 (0.5)	53 (1.8)	95 (0.9)

[a] Not mutually exclusive categories
[b] Defined using the Pediatric Age-adjusted Shock Index (SIPA)

© 2024 Deshommes T et al. *JAMA Network Open.*

eTable 3. Subgroup analyses: relative and absolute frequencies and inter-hospital variation of low-value practices by age group, biological sex, and admission year for pediatric trauma admissions, 2016-2022

	Age (years)				Biological sex[a]		Date of admission	
**Low-value practice** Incidence; n per 1000 admission; ICC	**<1 yoa N=1072**	**1-4 N=2248**	**5-11 N=4434**	**12-15 N=2957**	**Female N=4066**	**Male N=6415**	**Apr2016-Mar2020 N=7318**	**Apr2020-Mar2022 N=3393**
Head CT in children at low risk on the PECARN CT head rule	22.5; 88.6; 22.2	5.5; 34.7; 14.0	5.8; 23.2; 8.4	5.5; 25.4; 9.7	7.0; 32.0; 5.8	7.1; 33.2; 7.0	7.1; 32.8; 8.2	7.1; 32.7; 13.6
Cervical spine CT in children negative on the PECARN cervical spine rule	1.1; 7.5; 22.5	0.9; 4.0; [e]	1.7; 8.8; [e]	2.7; 12.2; 16.5	1.2; 6.1; 22.0	2.0; 10.3; 0.0	1.7; 8.6; 5.1	1.7; 8.5; 16.7
Abdominal/pelvic CT in children at very low risk on the PECARN abdominal injury algorithm	0.4; 0.9; [e]	1.1; 1.8; 42.9	2.0; 1.8; 9.0	1.3; 1.0; [e]	0.6; 0.7; 63.7	1.9; 2.0; [e]	0.7; 0.8; [e]	3.0; 2.9; [e]
Use of whole-body CT in children	0.2; 1.9; [e]	0.4; 4.0; 49.6	0.5; 5.0; 35.6	1.9; 18.6; 10.2	0.6; 6.4; 0	0.9; 9.4; 19.8	0.7; 6.7; 24.4	1.2; 11.5; 13.0
Pretransfer CT in children with a clear indication for transfer[b]	60.0; 2.8; [e]	71.4; 4.4; [e]	77.3; 3.8; 98.9	59.3; 5.4; 21.4	55.0; 2.7; 0.4	73.3; 5.1; 14.9	68.3; 3.8; 21.5	66.7; 5.3; [e]
Post-transfer repeat CT in children without clinical deterioration[c]	9.7; 2.8; [e]	20.0; 3.1; [e]	11.3; 3.2; [e]	12.9;6.4 ; 8.7	11.0; 3.0; 9.6	13.5; 4.7; [e]	11.1; 3.4; [e]	16.1; 5.3; 6.6
Repeat head CT in children without clinically significant intracranial lesions or clinical deterioration	0.0; 0.0; [e]	0.0; 0.0; [e]	0.6; 0.9; [e]	0.6; b;96.9	0.3; 0.7; 96.4	0.2; 0.5; [e]	0.2; 0.5; [e]	0.4; 0.9; [e]
Neurosurgical consultation in children without clinically significant intracranial lesions[d]	17.6; 42.9; 19.7	9.3; 12.0; 21.8	9.2; 7.2; 15.7	11.3; 10.1; 11.4	11.2; 12.3; 15.6	11.5; 12.2; 31.2	12.1; 13.1; 13.5	10.6; 11.5; 25.6
Hospital admission in isolated blunt abdominal trauma with a negative CT	44.4; 3.7; [e]	17.9; 5.3; [e]	11.3; 6.1; 18.7	5.3; 3.7; 20.3	11.9; 5.9; 16.9	9.0; 4.4; 18.7	10.0; 4..9; 31.0	11.1; 5.3; 34.1
Hospital admission in isolated mild TBI without clinically significant intracranial lesions	33.4; 233.2; 11.8	40.2; 132.1; 12.2	43.8; 74.7; 17.3	37.0; 57.2; 18.4	41.9; 107.5; 10.5	37.2; 90.6; 13.3	40.2; 105.1; 12.8	35.4; 81.9; 14.0
ICU admission in isolated TBI without clinically significant intracranial lesions	1.4; 6.5; 90.8	1.5; 4.9;91.5	2.1; 3.6; 95.9	4.7; 6.8; 54.0	2.4; 5.7; 93.5	2.1; 4.7; 92.3	2.3; 5.3; 92.9	2.1; 4.4; 91.8
Surgical management in children with solid organ injury who are hemodynamically stable	0.0; 0.0; [e]	1.9; 0.4; [e]	2.9; 1.1; [e]	2.5; 1.4; 92.9	3.0; 1.0; b	2.3; 0.9; b	1.8; 0.7; 95.6	3.8; 1.5; [e]

© 2024 Deshommes T et al. *JAMA Network Open.*

Angiointervention in hemodynamically stable children with low grade (I-III) solid organ injury	[e]	[e]	[e]	[e]	[e]	[e]	[e]	[e]
DVT prophylaxis in prepubertal children	2.2;1.9;0	11.5;4.4;0.1	6.0;2.0;0	NA	4.4;1.2;10.6	7.7;2.3;0	4.1;1.1;0	9.8;3.8;0

Low Moderate High

CT, computed tomography; ICC, intraclass correlation coefficient; ICU intensive care unit; NA, not applicable; PECARN, Pediatric Emergency Care Applied Research Network; TBI, traumatic brain injury
[a]Missing for 230 patients (2%)
[b]Applies to level III and IV referral centers
[c]Applies to pediatric trauma centers and level I-II adult trauma centers
[d]Consultation in the emergency department for neurosurgical centers (level I and II) and transfer to neurotrauma trauma centers for level III/IV centers
[b] Could not be estimated due to low sample sizes

© 2024 Deshommes T et al. *JAMA Network Open.*
