# Supplementary material for: Low-Value Clinical Practices in Pediatric Trauma Care
Source: JAMA Netw Open. 2024 Oct 29;7(10):e2440983. doi: 10.1001/jamanetworkopen.2024.40983 (PMC11522939; doi:10.1001/jamanetworkopen.2024.40983)
Supplement: Supplement 2. — Data Sharing Statement [file jamanetwopen-e2440983-s002.pdf]

**Data Sharing Statement**

**Data**
**Data available:** No

**Additional Information**
**Explanation for why data not available:** The data cannot be made available because of data protection laws but the programs used to conduct statistical analyses can
